# Body map stories from Colombia: experiences of people affected by leprosy and the influence of peers during diagnosis and treatment

**DOI:** 10.1186/s12939-024-02152-0

**Published:** 2024-05-13

**Authors:** Martha Cecilia Barbosa Ladino, Camila Jiménez Betancourth, Lucrecia Vásquez Acevedo, Melanie Haag, Janina Zirkel, Eva-Maria Schwienhorst-Stich, Miriam Navarro, Christa Kasang, Ildikó Gágyor, Sandra Parisi

**Affiliations:** 1 DAHW Latin America, GLRA German Leprosy and Tuberculosis Relief Association, Bogotá, Colombia; 2 FELEHANSEN National federation of people affected by Hansen`s disease in Colombia, Bogotá, Colombia; 3 Department of General Practice, University Hospital Würzburg, Würzburg, Germany; 4 Internal Medicine, Unit of Infectious Disease, University Hospital Würzburg, Würzburg, Germany; 5 Epidemiology Unit, Public Health Centre of Elche - FISABIO, Alicante, Spain; 6 Department of Public Health, Science History and Gynaecology, Universidad Miguel Hernández, Alicante, Spain; 7 DAHW HQ, GLRA German Leprosy and Tuberculosis Relief Association, Würzburg, Germany

**Keywords:** Hansen`s disease, Body map stories, Leprosy, Life experience, Peer support, Latin America, Diagnostic delay, Self-management, Prevention of disability, Thematic analysis, Qualitative research

## Abstract

**Introduction:**

Almost one third of people affected by leprosy in Colombia suffer from disability, which often results from delayed diagnosis and treatment. We aimed to explore the experience of people affected by leprosy during the process of diagnosis and treatment and if and how this experience was influenced by peers.

**Methods:**

A qualitative study using body map stories was conducted from October 2019 to February 2020 in Colombia. Adult people affected by leprosy were recruited through patient associations in different cities. We conducted three sessions with an average duration of 2–3 h per participant, during which the participants created a painted map of their body and chose symbols to represent their experience, while being engaged in an informal interview. The sessions were audio recorded, transcribed verbatim and analyzed thematically by an interdisciplinary team, consisting of physicians, social workers and a person affected by leprosy.

**Results:**

The 17 study participants (11 female) were aged 20 to 70 years. Leprosy-related manifestations ranged from no to advanced disability. Some participants were active members of associations for people affected by leprosy. Three main themes were identified during analysis: (1) A long pathway to diagnosis, (2) Therapy as a double-edged sword and (3) The influence of other people affected by leprosy. The participants described an often years-long process until being diagnosed, which was marked by insecurities, repeated misdiagnosis, and worsening mental and physical health. Delayed diagnosis was related to late health care seeking, but also to inadequate health communication, lack of leprosy-related knowledge and negligence among health care workers. A high desire to cure motivated the participants to take their medication rigorously, despite the high treatment burden. Support from peers, either within the own social environment or provided from associations, contributed to a faster diagnosis and increased therapy adherence. Peers helped to recognize the symptoms, urged patients to seek care, recommended physicians with leprosy-related knowledge and provided a realistic example of both disease severity and curability.

**Conclusion:**

People affected by leprosy experience a significant burden during the process of diagnosis and treatment. Involving well-trained peers could foster early diagnosis, treatment compliance and prevention of disability.

**Supplementary Information:**

The online version contains supplementary material available at 10.1186/s12939-024-02152-0.

## Introduction

Leprosy, also known as Hansen’s disease, is a slowly progressing chronic disease leading to sensory loss and resulting disability if no adequate measures are undertaken. Early diagnosis and treatment can cure the disease, prevent disability and halt transmission [1]. Despite official elimination as a public health problem in most countries, around 2 million people are currently living with disability related to Hansen`s disease worldwide, affecting both old and newly diagnosed cases [2, 3].

In Colombia, the average number of new cases diagnosed per year ranges between 350 and 400. It is estimated that there are around 2.000 people living with related disabilities. Colombia has a national program for leprosy, that -in its official post elimination stage- struggles with limited financial and human resources, especially at the regional level responsible to implement national guidelines [4]. There is a significant delay of diagnosis resulting in a disability rate of approximately 30%, including sensory loss (grade 1 disability) and visible deformities (grade 2 disability) [4–6]. The latest report of the national program highlights that the current rate of grad 2 disability (0.83 per million inhabitants), which is often used as a proxy marker for diagnostic delay, still exceeds the national goal (0.58 per million inhabitants) [7]. Moreover the presence of newly diagnosed cases in children under 15 years indicates ongoing transmission [7]. A quantitative study identified inadequate knowledge among health care workers (HCW), multiple consultations before diagnosis and delayed seeking of health care as the main reasons for delay in diagnosis [5].

A survey among Colombian people affected by leprosy (PAL) reported that 49% suffered from mental distress, 27% from participation restriction and 52% from significant stigma [8]. A recent review exploring the lived experience of PAL described a multi-domain effect on affected individuals that influenced their help-seeking behavior and adherence to treatment. Social exclusion and stigma were important aspects reported [9]. Although the analysis only included articles in English, the systematic literature search was conducted without language restrictions and did not identify any studies published in Spanish. To the best of our knowledge, there are no qualitative studies from Colombia exploring the lived experience of PAL. Their experiences during the process of diagnosis and treatment might help to better understand the underlying phenomena leading to those severe psychosocial consequences and the high grades of disability, since early diagnosis, treatment compliance, management of leprosy reactions and individual prevention of disability are considered important to prevent chronic disability [10, 11].

Since 2008, with the support of GLRA *German Leprosy and Tuberculosis Relief association* (GLRA), a step wise self-organization of PAL in Colombia led to the creation of 11 patient’s association and the national federation FELEHANSEN. Nowadays, around 700 PAL and family members are officially associated to these associations and self-help groups. Apart from building support structures and implementing projects covering different domains such as livelihood, education and health access to improve the social determinants of health, these associations are an important component of Hansen`s disease control, supporting the national program in early diagnosis, contact management and follow up of treatment. The influence of peers on the experience of other PAL and on early diagnosis and treatment has however never been assessed.

Body map stories (BMS) are a visual and arts-based methodology that offers “new ways to engage with participants on a more equal footing, thereby promoting participant engagement and empowerment” [12]. BMS produces rich, contextual insights and helps to explore sensitive and controversial topics that can be difficult to articulate verbally [12, 13]. BMS helps to understand complex situations, determinants of health and internal and external factors that might influence the experience and decisions of participants [12]. It has been used in exploring coping strategies and support systems of other diseases, such as mental health problems [14], HIV, disabilities and health aspects related to migration [12, 15, 16], but to our knowledge has never been applied to explore the narratives of PAL and only once in other neglected tropical diseases (Chagas disease, publication pending).

The aim of our study was to use BMS to explore the experience of PAL during the process of diagnosis and treatment, as well as the underlying phenomena of delayed diagnosis, treatment and potentially resulting disability. We furthermore wanted to explore if and how this experience was influenced by peers.

## Methodology

A qualitative study using BMS was conducted from October 2019 to February 2020 among PAL in Colombia. BMS is a technique where participants use drawings, cut printed images from journals or use other media to visually represent their experiences on their life-sized body silhouette, while engaging in a constant conversation with the researcher [17]. The methodology was chosen due to its usefulness to explore topics that are difficult to explain or are stigmatized and its accessibility including for vulnerable populations such as those with low levels of literacy [14]. BMS can be both, a research process and product [18].

### Study population and setting

The study population consisted of adults (above 18 years) diagnosed with Hansen`s disease, including newly diagnosed cases and those who have completed treatment. Both active federation/association members and PAL that had never participated in any activities by associations or GLRA were invited to participate. Exclusion criteria were residing outside Colombia and not having the time to participate on three consecutive days.

Participants were recruited through the members of FELEHANSEN/local associations until no new themes were identified and data saturation was perceived within the research team. The interviews were conducted by two social workers (MB, CJ) from GRLA and one PAL (LV, legal representative of FELEHANSEN) in the respective communities, either at the associations´ meeting sites, or, if preferred, at the participants’ home. Whenever possible, study participants were recruited purposefully in order to assure a broad range of participants from different Colombian sites, with diverse grades of disability, educational status and rural/urban living situation. Active federation members, however, had to contact the study team if they wanted to take part. This procedure was chosen to assure that only participants that really wanted to share their lived experience were included and that no moral obligation was felt due to the close relation of GLRA and leaders.

### Data collection

The BMS consisted of three sessions per participant, each with a 2–3-hour duration. The focus of the sessions was (1) life before the disease, (2) living with the disease (3) relevant supporting structures and one’s own competences, strengths and coping mechanisms. A semi-structured interview guide was used (see additional files 1 and 2, adapted from Gastaldo et al. [17]). The first session started with an individual in depth interview of approx. 30 min on their experience with the illness. The participants were then asked to create their individual BMS with colors, images, collages and writings. During the whole process the facilitator and participant were engaged in a non-formal dialogue about the participants’ experiences and explaining the meaning of chosen images, colors and other representations.

### Data analysis

The recorded interviews were transcribed verbatim and any identifiable information was removed. Thematic analysis was performed on the written transcripts which included the verbal descriptions and own interpretations of participants on their drawings and symbols used during the BMS sessions [14, 18–20]. The visual elements were not analyzed separately but only used to highlight the participants’ descriptions.

We focused on manifest content and used a realist approach which means that the experiences, meanings and lived reality of participants are reported and reflect an assumed reality within the data [20]. We conducted an overall thematic description of the data, which is suitable to explore the experience of an under-researched population [20]. In order to present the findings with enough depth, this manuscript focusses on the experiences during the process of diagnosis and treatment, whereas the topic of adapting to a life with Hansen`s disease will be published elsewhere.

The coding team consisted of 5 people (2 Colombian social workers, 1 Colombian PAL, 1 German physician (Spanish native), 1 German medical student (fluent in Spanish)). The inclusion of multiple coders with a mix of relevant experience can help to increase the quality of analysis by mitigating the effect of individual coder`s perspectives and backgrounds [21]. The Colombian team member (MB, CJ, LV) had pre-established relationships with several participants due to previous involvement in local GLRA/ FELEHANSEN activities. This helped to create an atmosphere of trust during interviews and to contextualize the results. The participation of non-Colombian coders (SP, MH) allowed to identify and reflect context specific aspects in comparison to other countries and added expertise in thematic analysis. It furthermore allowed to better reflect and discuss how the previous relationship between interviewers and participants affected the answers. It was for instance decided to explore general peer involvement, including friends and families, rather than the influence of associations on the participants` experience.

Initial codes were identified jointly through team discussion [22], after each member read at least 3–4 transcripts, while 3 researchers were familiar with all transcripts. Those initial codes were mainly based on the chronological order of experience [23], research objectives and initial themes inductively identified. Both, Microsoft Word and NVIVO12 software [24] were used for coding. A table was constructed, which entailed the overall themes and subthemes together with corresponding data [19] (see also additional file 3 and 4 for verbatims), which was discussed and adapted during regular online meetings. These meetings also served to discuss the overall progress, difficulties arising during the coding process as well as the meaning and implication of findings. Intercoder reliability was assessed by consensual coding. Case summaries were performed as an intermediate step of analysis to gain an overview of individual participants’ perspectives on important themes. Thematic maps also served to organize and refine identified themes [14, 20]. Additional file 5 shows an initial thematic map exploring the underlying phenomena for high grades of disability. We used SRQR guidelines to report our findings (additional file 6) [25].

### Ethical aspects

The project was granted permission by the ethical committee of the Sanatorium of Contratación, Santander, Colombia. All participants were adults, providing written informed consent. The study was participative from the beginning and discussed in its initial planning phases with the federation FELEHANSEN. A PAL and federation member (LV) was involved as co-investigator during all phases of the study, from the planning, recruitment, capacity building, data gathering, analysis to publication and dissemination of the results. The publication draft was furthermore shared with other participants to assure that they perceived the results as representative of their experience.

Confidentially was discussed in depth with the federation, especially because some leaders did not want to use pseudonyms. They considered giving their testimony an important step of their healing process and did not want to hide their identity. We decided jointly, that participants could choose their name, but we would not display which names are real (e.g., middle names) or fictive. Considering that the leaders know each other, we will not include any personal information that can be linked to the individual (e.g., name of towns). It was also discussed to use both terms, Hansen`s disease and leprosy, which is associated with stigma, but better known, in order to target a wider audience and because some participants identified more with being a “person affected by leprosy”. At the end of each session the participants were asked how they felt. In case of need, participants had the possibility to be attended by an interdisciplinary team, including physicians and/or psychologists from GLRA and the regional program, which was however not necessary. Financial support was provided to FELEHANSEN, because some participants wanted to organize a visual BMS exposure to share their testimony with a wider audience on World Leprosy Day 2023.

## Results

A total of 17 BMS were conducted in different towns of Colombia such as Bogotá, Cartagena, Cúcuta, Cali, Contratación, Neiva y Villavicencio. BMS participants came from both rural and urban areas and were 20 to 70 years old (Table 1). Most participants had already finished treatment but several were affected by chronic disability and two participants reported suffering from severe, recurring reactions. During the exercise, the participants became more confident in expressing their ideas and described a liberating experience, telling stories that they had never been able to share before.

*At the first moment you might not know how to express what you feel, but later you start developing ideas (…) it made me see things and it made me look at my life differently. (Flor del Campo)*

*You release pent-up emotions, you take a weight off your shoulders. (Angie)*

**Table 1:** Study characteristics of included participants

Pseudonym	Gender	Age category*	Level of education	Treatment	Grade of disability	Involvement in association/ GLRA activities
Lázaro	Male	51 to 60	Incomplete Primary School	Completed	2	Leader
Luchador Incansable	Male	51 to 60	High school diploma	Completed	1	Leader
Angie	Female	20 to 30	High school diploma	Ongoing	0	Not involved
Hela	Female	41 to 50	High school diploma	Completed	2	Leader
Yamileth	Female	20 to 30	High school diploma	Completed	0	Participant
El Negro	Male	31 to 40	High school diploma	Completed	2	Not involved
Chamo	Male	31 to 40	High school diploma	Completed	2	Participant
Flor del campo	Female	41 to 50	High school diploma	Completed	2	Not involved
Pequena rosa	Female	61 to 70	High school diploma	Completed	0	Not involved
Palomita	Female	41 to 50	High school diploma	Completed	2	Leader
Carlos	Male	61 to 70	Incomplete Primary School	Completed	2	Not involved
Chiqui	Female	41 to 50	High school diploma	Completed	1	Leader
María	Female	20 to 30	Superior studies	Ongoing	0	Not involved
Mariposita	Female	51 to 60	Primary School	Completed	1	Not involved
Leticia	Female	61 to 70	Incomplete Primary School	Completed	2	Participant
Saray	Female	31 to 40	High school diploma	Completed	1	Leader
Daniel	Male	51 to 60	High school diploma	Completed	0	Participant

* Age was only collected in categories, to prevent the identification of participants

Three main themes were identified (Table 2): (1) a long pathway to diagnosis (2) therapy as a double-edged sword, (3) The influence of other people affected by leprosy.

**Table 2:** Coding Framework

**A long pathway to diagnosis**
Unimpressive discrete symptoms • A non-hurting skin lesion doesn´t affect me in anything • Using self-medication for common skin diseases Failure of the health care system to diagnose Hansens`s disease • Facing general health access barriers • One in a million of HCW experienced in Hansens`s disease • Negligent work of HCW Suffering during the search for diagnosis • Wandering from doctor to doctor • Fear of symptoms, not knowing what is happening • Lack of professional therapy leads to desperate self-therapy and self-mutilation • Inappropriate communication of HCW causes negative emotional reactions • Misdiagnosis damages life and health The key to diagnosis • Screening and active case finding as fast detection methods • “Having luck” to find Hansen-experienced HCW • Diagnosis due to differential diagnostic procedure • Fighting for one`s health, perseverance and knowledge about Hansen`s disease • Support and pressure by social network • Person facilitating the diagnosis perceived as divine being
**Therapy as a double-edged sword**
Therapy as the beginning of recovery • Being reassured of cure • The will to persevere therapy to get healed • Good treatment does not just mean taking pills Therapeutic burden • Body-insecurities and discrimination, being “visible” for everyone • Irritation caused by changing color of body fluids • Harsh adverse reactions to medication drain off last power reserves • Dealing with multiple appointments and medical infrastructure • Living in the uncertainty of being healed Not taking medication and other preventive action • Frustration about never-ending treatment, “Now it is too late anyway” • Cluelessness about function of treatment • Negligence with preventive measures • Not being able to comply, being trapped in the socio-economic cage
**The influence of other people affected by leprosy**
Peers enabling diagnosis • Contact tracing as a fast detection method • Leading to the right places • Conviction that it has to be leprosy • Becoming the helping peer to prevent others from diagnostic delay Experience of peers fosters therapy adherence • Understanding of severity through peer support • Experiencing curability Peers give advice and orientation about living with the disease • Advice of non-peers is not sufficient • Peers as examples and teachers • Sharing stories makes one feel less alone

### Theme 1: a long pathway to diagnosis

#### Unimpressive discrete symptoms

The disease often started with an inconspicuous rash or general malaise which were not taken seriously by the study participants, who therefore did not seek medical help (VS1, see verbatims in additional files 3 and 4). In some cases, own disease theories led to self-therapy (VS2), such as antifungal creams or the discontinuation of contraceptives, which were blamed for an allergic reaction. Since the participants lacked knowledge about the clinical presentation of Hansen`s disease, they assumed more common diseases to be responsible for their symptoms leading to self-management and delayed health care seeking.

#### Failure of the health care system to diagnose Hansen`s disease

The narrations on diagnostic delay predominately centered around medical undersupply and inadequate care. While some reported late health seeking behaviour due to long distances when living in rural areas or in the periphery of large cities (VS3), others experienced difficulties in making a timely appointment. Having money and contacts for private medical appointments accelerated this process (VS4). A common issue identified was the lack of experience and knowledge about Hansen`s disease among HCW (VS5-6). Hansen`s disease related lesions were repeatedly misdiagnosed for allergies or mycosis. While the study participants acknowledged that the diagnosis of Hansen`s disease is not easy to make (VS7) and requires careful examination and differential diagnosis, they also reported negligent work of HCW (VS8), which at times denied considering Hansen`s disease despite being told about the suspicion and family exposure. Moreover, some patients were confronted with severe diseases like cancer without any further examination or explication (VS9).

*Then they told me that it was leishmaniosis, the third diagnosis was cancer, the fourth syphilis and the fifth AIDS. (Lázaro)*

#### Suffering during the search for diagnosis

The phase prior to diagnosis was often described as a sequence of appointments and examinations (VS10) meanwhile allowing the disease to proceed and letting them feel physically exhausted (VS11). Feeling left alone and insecure, the participants did not know what was happening in their bodies and some felt the urge to become active and take action against their symptoms to find relief, which, due to the loss of nociception, could result in self-mutilation (VS12). Inadequate HCW-patient-communication was a recurring theme. Patients were not taken seriously, they got confused by insufficient clarification, frightened by exaggerations, or felt discriminated (VS13-14).

*Because people are discriminated within the health sector. When a professional trained in the subject, the illness (referring to Hansen`s disease) arrives, things change a little, but in the first instance the discrimination is great in the health sector, the stigma is great. (Luchador Incansable)*

Misdiagnosis also led to direct consequences. One patient received an immunosuppressive therapy for reactive arthritis which enabled Hansen`s disease to prosper (VS15), while another participant was kicked out of his house after being misdiagnosed with highly stigmatized AIDS, which completely deprived him of his social network (VS16).

#### The key to diagnosis

The circumstances of diagnosis seemed to depend mainly on luck. Only few people got tested through active case finding (VS17) and therefore received a timely diagnosis. While some participants were lucky to contact Hansen`s disease experienced HCW early (VS18) or a doctor who conducted good differential diagnostic examinations (VS19), others had to fight for their health looking for repeated medical attendance (VS20). In some cases, third persons like friends or family members with or without knowledge about Hansen`s disease played a crucial role in attaining the diagnosis by urging the participants to seek health care (VS21). The person who finally facilitated the diagnosis was at time described using words such as “angel” or “guardian angel”, reflecting the religious beliefs of participants but also their relief of being diagnosed and the high psychological burden during the diagnostic process. The importance of religious expressions and images was particularly noticeable to the non-Colombian scientists.

*I already had been in there for some time (referring to a health facility) and I continued to lose sensibility and the numbness was spreading. I nurse came by my room, I saw that she wasn´t going to come in…she was like, like the Guardian angel. She said that she was of a special program… she told me that I had to go to XX (name of a town). (Chiqui)*

### Theme 2: therapy as a double-edged sword

#### Therapy as the beginning of recovery

The therapy was broadly appreciated as the measure to cure the disease (VS22) offering hope and helped to adjust to the diagnosis. The will to cure and knowledge about the severity of Hansen`s disease motivated the participants to take their medication responsibly (VS23). Participants furthermore described that a good therapy does not only entail medication but also self-protective behaviour, healthy alimentation and rest (VS24).

*When I started to get treatment I realized that you can be healed (Palomita).*

#### Therapeutic burden

**Fig. 1:**
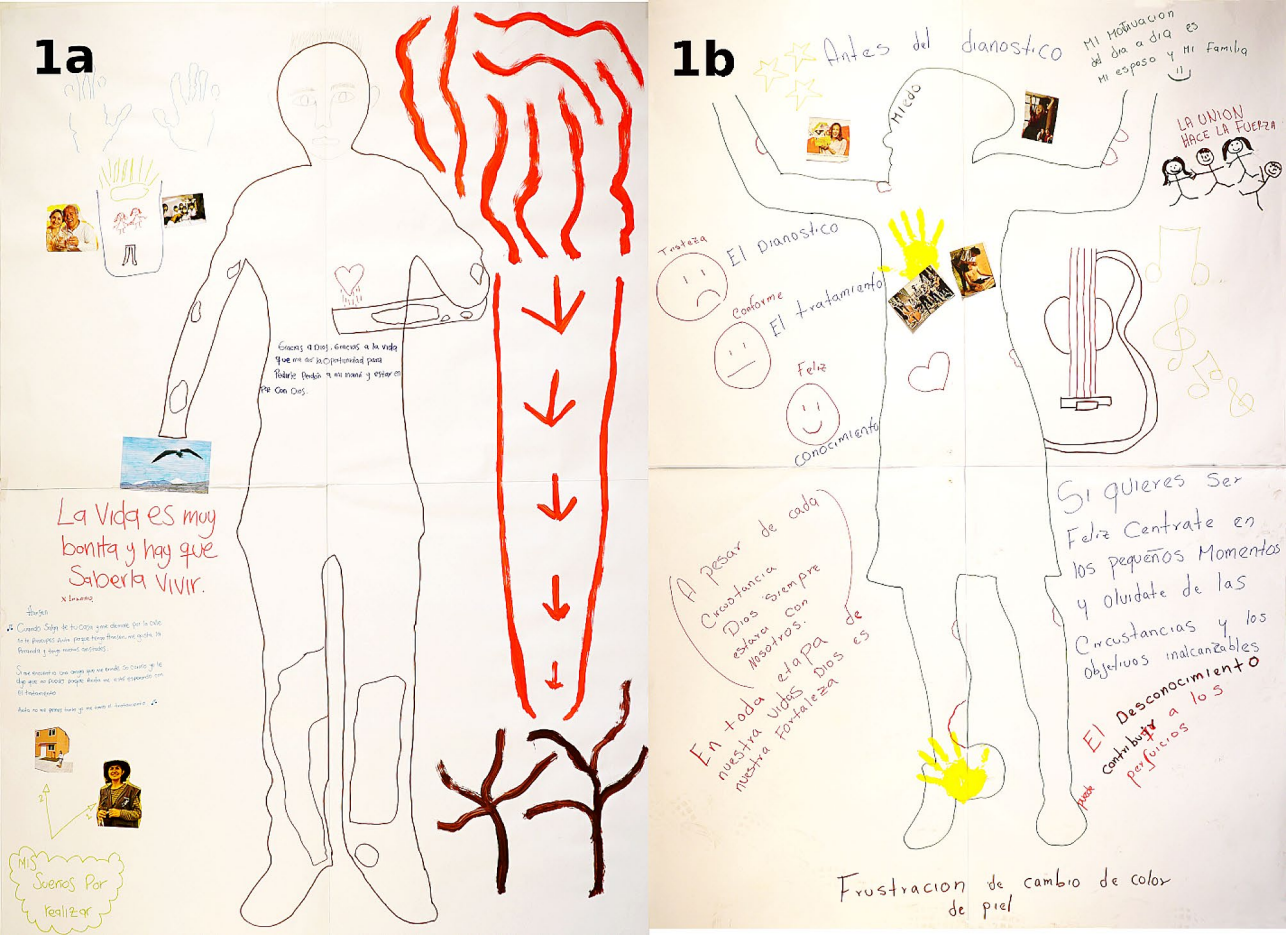
Body maps of Lázaro and Yamileth. **a**) Body map of Lázaro, who decided to paint his silhouette with a brownish color, because people used to call him “Cafecito” due to Clofazimine side effects of coloring the skin lesions darker. **b**) Body map of Yamileth, depicting her fear (forehead) and worries during the diagnostic process, her slightly improved emotional health during treatment and her frustration due to skin color changes

The participants were thankful for the availability of free treatment, but highlighted its unpleasant side effects. The Clofazimine-triggered skin hyperpigmentation was described as unfamiliar and stressful by almost all participants (Fig. 1). It made the disease visible to everyone and led to unpleasant questions, especially since many participants did not like to talk about the disease (VS25) fearing discrimination and rejection (VS26).

*I stopped to go out because of the explanations: why I don´t have eyebrows? Why I am tanned? Or what is happening to me? Because I was fine and I remember how I felt bad and I started to cry. And, to not give too much explanation I stayed in my house. (Chiqui)*

Less apparent and therefore less burdening was the red coloration of body secretions caused by rifampicin triggering more surprise than shame (VS27). Severe adverse reactions such as hepatic and renal damage (VS28) were also described. The time during therapy was further complicated by the uncertainty of being healed (VS29) and by the challenging task to manage the own disease, to deal with the chaotic health system and to try to fit appointments into one´s daily life (VS30).

#### Not taking medication and other preventive action

Despite these hardships mentioned by almost all participants, only few reported breaks from medication and inconsequent fulfilment of preventive measures. While belief in treatment as cure promoted compliance, doubting in treatment efficacy and frustration about its never-ending could trigger the opposite behaviour (VS31). Also, insufficient information provision could lead to repeated starting and stopping of medication (VS32) while negligence with preventive measure could lead to injuries. Some participants explained that socioeconomic barriers kept them from further engaging in healthier preventive behaviour, such as fulfilling recommendations by nutritionists (VS33) or hygiene standards.

*So, when I took of my cloths it started bleeding, because I had ulcers and wool sacks, and (the doctor) asked me: “What is this?” And I told him “It`s toilet paper”. And he told me: “How could it occur to you using this?!” So I told him “Well I don´t have the money to buy bandages.” (Lazaro)*.

### Theme 3: the influence of other people affected by leprosy

#### Peers enabling a fast diagnosis

Other PAL (either within the family/community or from the association) facilitated and accelerated the diagnosis through routine testing of their surrounding (VS17) or by recommending certain medical institutions and doctors with whom they made good experiences (VS34). Sometimes, due to prior experience, friends and family indicated on Hansen`s disease and urged the participants to seek adequate medical treatment.

*Since I have relatives, uncles, aunts and cousins with leprosy, my friend told me “you must have leprosy because of this pain you have and the medication doesn´t work neither”, so she told me to go and I didn´t do so (…) so time passed until one day she scolded me and said: “Sister, what are you waiting for? That you get leprosy? That you will be left in pieces? I am tired of telling you to go to a leprosy doctor (…)”. (Mariposita)*

There was however also a diverging case with delayed diagnosis despite typical clinical signs and the involvement of peers.

#### Being oneself the helping peer

An interesting point identified was the observable role change that the study participants underwent. While initially uninformed and helpless during the diagnostic process, they later developed the need to help others in the same situation as they were at the time. Thus, they directly informed their environment about the disease (VS35) or - if restricted by shame - at least scanned their family for symptoms in order to prevent a delayed diagnosis with its negative consequences.

*Because I haven’t told them. For fear of rejection. So they won´t feel bad. But I am sure that my daughter is not ill because I have seen her well. I always check that she has nothing, but she is fine. She has a normal life so far. (Daniel)*

#### Experience of peers fosters good therapy adherence

**Fig. 2:**
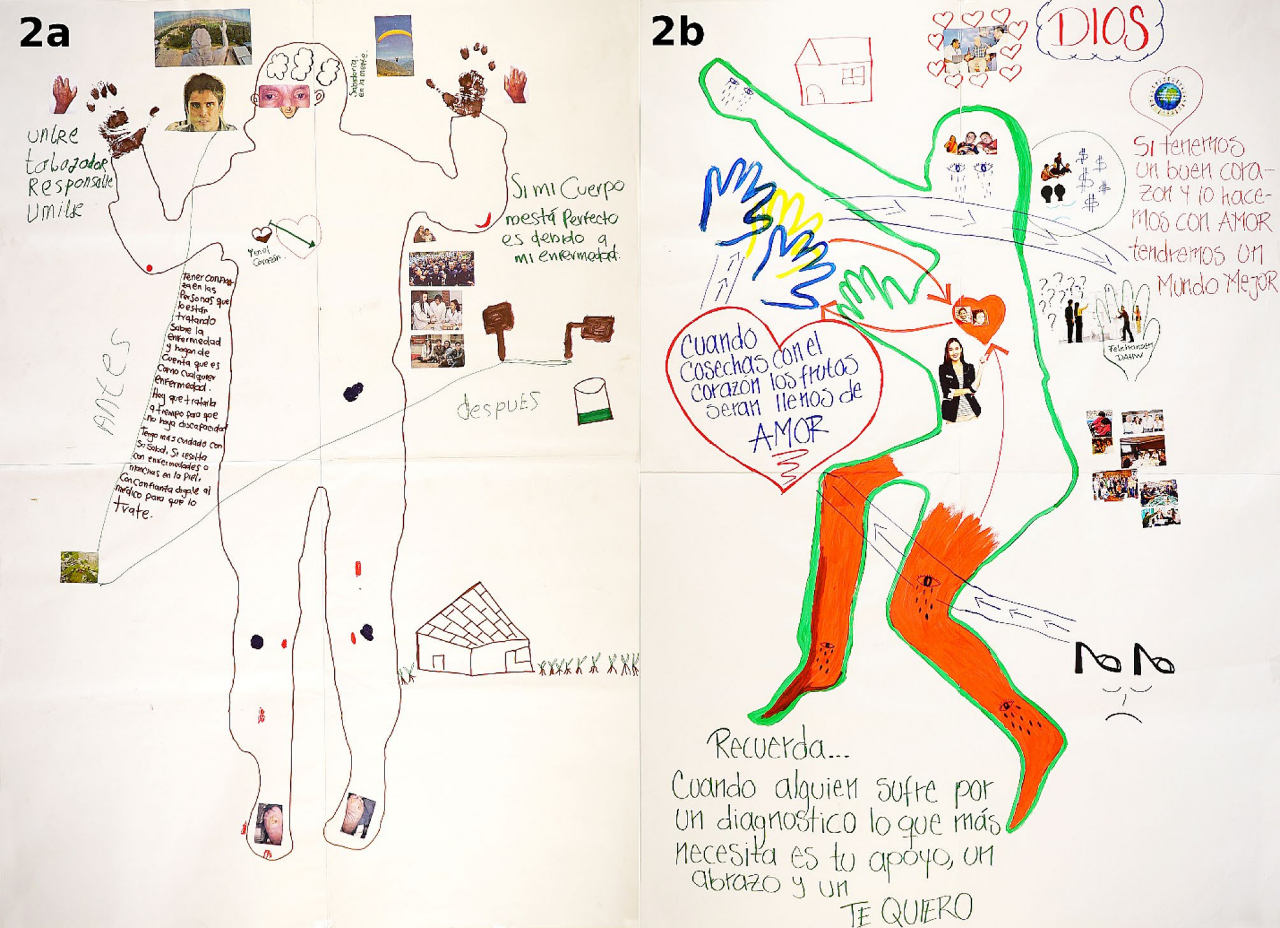
Body maps of Daniel and Saray. Examples of how the participants represent their disability and their message to help others. **a**) Body map of Daniel, identifying himself as a humble, responsible and hardworking man. He wrote “if my body is not perfect, it`s due to my disability” and chose to use photos of feet ulcers. In his message to the public he emphasizes the importance of treating leprosy such as any other disease, seeking diagnosis and treatment early, trusting in HCW advice and engaging in preventive behavior. **b**) Body map of Saray, showing crying eyes and sad faces due to the disability in her legs, impeding her to use high heels. She depicts how her question marks were met by a helping hand by other peers from FELEHANSEN. Her message to the public says: Remember… If someone is suffering due to a diagnosis, what this person needs most is your support, a hug and a “I like/love you”

Knowledge about Hansen’s disease could also lead to enhanced therapy adherence (VS36). Through the contact to peers, the participants could assess the severity of the disease and meanwhile understand that you can be cured even though one can suffer permanent disability (Fig. 2).

#### Peers give advice and orientation about living with the disease

The contact to other PAL was perceived as beneficial and relieving: Especially in the period after diagnosis, the participants faced fears and worries, lacking clarification by someone they could trust. Even though HCW informed and their social network tried to comfort, the participants perceived this as insufficient to adapt to the disease. By receiving information from peers, the participants experienced hope and orientation (VS37) since they provided a good and realistic example of how to surmount the disease.

## Discussion

This qualitative BMS study is the first of its kind to examine in depth the experience of PAL in Colombia during the process of diagnosis and treatment. Our results highlight that a long pathway to diagnosis is not only related to late therapy, irreversible disabilities and ongoing transmission, but also to psychological and leprosy-independent health damage caused by experiences during the diagnostic process. The involvement of peers significantly influenced the experiences and impacted on early diagnosis and treatment initiation, as well as treatment compliance.

### The process of diagnosis

The experienced long path to diagnosis coincided with worldwide studies on diagnostic delay [3, 5, 26]; Hansen`s disease is often found after many years and therefore with already existing disabilities [3, 27, 28]. In a quantitative study from north-eastern Colombia, the mean delay in diagnosis has been reported to be 33.5 months [5]. In line with other studies worldwide, we found delayed health service seeking behaviour [28–30], general health access barriers, self-medication and misdiagnosis by health workers [27] as reasons for a long process to diagnosis. Our study highlights the importance of misdiagnoses by HCWs, which indicate poor training [31], but can also be caused by medically negligent work [31]. Other authors previously described this as a major problem and concluded by calling for better education and training, especially for HCWs [3, 27, 29, 30, 32–34].

Our study shows that the long diagnostic process does not only lead to disability [35], but also directly causes psychosocial damage. The study participants described suffering severe consequences until finally receiving their diagnosis. This time span was marked by health concerns, which sometimes resulted in self-therapy and - due to the lack of nociception and the lack of professional help - even self-mutilation. Frightening to discriminatory behaviour by HCWs and misdiagnosis also contributed to the mental health impact previously described in PAL [36].

### Treatment experience

The experienced treatment burden is known to deteriorate mental health in PAL [37–39]. Especially Clofazimine-related hyperpigmentation was perceived as extremely distressing and was the life-defining aspect under medication. In line with other studies, PAL were afraid that they would be “outed” as leprosy patients due to their pigmentation [9, 40] fearing social stigma and discrimination [35], and this concern has previously been associated with incompliance [9, 41]. Despite the good efficacy of MDT, the development of new drugs should be continued to improve quality of life and therapy adherence [34].

Despite the harsh therapy, the will to be cured and the awareness of the severity of the disease led to a high willingness to take medication and to accept preventive measures and restrictions. The provision of adequate health education (e.g. including information of Clofazimine side effects as being temporarily [9]) can foster therapy adherence and self-care [42, 43].

### The influence of social determinants of health and peers

In line with other studies [44–46], our results show that adherence to therapy and prevention is also dictated by socio-economic conditions. Several study participants did want to, but could not afford additional measures such as healthy nutrition or bandages. Moreover, as observed in other countries [3], socio-economic aspects such as rural living favoured delayed diagnosis. The high treatment compliance can therefore not solely be attributed to a high willingness to cooperate, but supports the importance of continued provision of free and easily accessible MDT therapy.

Seeking peer support has been described as an important coping strategy to alleviate the treatment burden in young people with chronic conditions [13]. Although the WHO emphasizes the need for peer engagement towards the goal of zero leprosy [34], their involvement during the process of diagnosis and therapy has hardly been studied. In a recent systematic review on effectiveness of active case finding campaigns, only one study mentioned PAL facilitating community access [47]. The review compared community health worker campaigns versus campaigns led by Hansen`s disease specialists and concluded that while trained community health worker were less expensive and guaranteed better community access, they often lacked on experience about Hansen`s disease. Involving trained PAL in early case detection could bridge that gap, by combining disease experience with community access. They could for instance, specifically be trained to apply screening tools, such as the Leprosy Suspicion Questionnaire that also includes early neurological symptoms [48]. Our participants described that the involvement of peers contributed to receiving their diagnosis quickly, to find HCW with experience handling Hansen`s disease and to promote good therapy adherence. Moreover, the participants reported how peers were able to provide initial advice and emotional support to disoriented, affected persons and thus alleviated the psychosocial burden. Since early detection and early treatment play an immense role in reducing the burden of disease and breaking chains of infection [49, 50], our study helped to understand the mechanisms in which peers may have a positive impact towards the goal of leprosy elimination. Future studies are needed to further explore best practices in peer support interventions and their impact on early diagnosis and treatment.

### Strengths and limitations

The focus of the BMS design, as an arts-based participatory research method, was not only on collecting research findings, but also on processing what was experienced. During the course of the interviews study participants became successively competent in presenting their emotions on the BMS and expressing themselves. Through the BMS, participants began to look at their illness from certain perspectives and adjusted their opinions during the interviews, reflecting and getting to know themselves in a new way, which let many participants to feel grateful for the experience. The involvement of a PAL (now legal representative of FELEHANSEN) during data analysis also helped the rest of the multi-country and interdisciplinary research team to better understand and contextualize the findings. It moreover lead to direct practical implications, such as negotiations of FELEHANSEN with the Colombian national program to integrate psychotherapy within its care package.

The previously described therapeutic effect of the BMS [22] however comes with the limitation that it cannot be clearly distinguished if the expressed attitudes and insights were pre-existing or formed during the interview. Due to the retrospective data collection, a recall bias can also not be ruled out, especially if the participants had been diagnosed years ago. While we tried to sample individuals purposefully, for ethical reasons, active federation member had to contact the study team if they wanted to participate. This could have led to a selection bias and an overrepresentation of certain characteristics and themes. Another important aspect is, that the interviewers were known by active federation members. This helped the participants to open up and share difficult stories. We tried to carefully reflect if and how this previous relationship influenced the interviews and used several methods, such as the involvement of coders with a different background and a thorough analysis of diverging cases to mitigate these effects.

## Conclusion

Our study helps to understand the underlying phenomena of disability among PAL in Colombia. The reasons identified include late health care seeking behaviour and mistreatment by PAL themselves or by HCW, all based to a certain degree on insufficient knowledge about Hansen`s disease. These findings highlight the importance of health education and empowerment of PAL and support the demand to improve the teaching and upskilling especially of HCW within a decentralized “zero leprosy” strategy. Involving well-trained peers in identifying and accompanying PAL during the process of diagnosis and treatment could help to foster early diagnosis and treatment compliance. Further studies are needed to identify best ways and to quantify the impact of peer involvement.

### Electronic supplementary material

Below is the link to the electronic supplementary material.

Additional file 1: BMS Interview guide English translation

Additional file 2: BMS Interview guide Spanish

Additional file 3: Additional Verbatims English translation

Additional file 4: Additional Verbatims Spanish

Additional file 5: Initial thematic map exploring underlying phenomena of disability

Additional file 6: SRQR Guidelines for reporting qualitative research

## Data Availability

The dataset supporting the conclusions of this article is included within the article and its additional files 3 and 4. Further data are available on reasonable request emailing Parisi_S@ukw.de.

## Abbreviations

HCW: Health care worker
BMS: Body map stories
PAL: People affected by leprosy
WHO: World health organization
