# Supplementary material for: Body map stories from Colombia: experiences of people affected by leprosy and the influence of peers during diagnosis and treatment
Source: Int J Equity Health. 2024 May 13;23:98. doi: 10.1186/s12939-024-02152-0 (PMC11092158; doi:10.1186/s12939-024-02152-0)
Supplement: Supplementary file 1 — Additional file 1: BMS Interview guide English translation [file 12939_2024_2152_MOESM1_ESM.docx]

**PRE INTERVIEW**

Reading and signing of informed consent form. Personal interview about their experience with Leprosy (35 min).

Semi-structured interview questions:

- I would like you to tell me about your experience with the disease....

- Do you prefer that we speak of the disease as Leprosy or as Hansen's? Why?

- Did you know anything about this disease before you knew you had it? What did you think

about it?

- Who told you that you had Leprosy/Hansen's? How did the person do it? Did you

understand? Did you have questions? Were they answered? Do you feel you know about

your disease?

- Did you know at the time what it meant to have the disease?

- What did the diagnosis of the disease mean to you? How has it affected you physically? And

emotionally?

- Has it affected your day-to-day life, in what way?

- CBR dimensions: how has it affected your health, education, social/family life, work,

community participation?

- Do you believe you are still fulfilling your duties?

- Has it affected your family relationships or your relationship in the community? Who have

you told about your illness?

- How have you adapted to your illness?

- Who has supported you in living with your illness?

- What resources have helped you the most?

- Have you had contact with associations (e.g. Felehansen) or other support groups? Tell us

about your experience!

**SESSION 1: BEFORE AND AFTER KNOWING I HAVE THE DISEASE**

Introduction to BMS (5 min)

Importance of what is told, not of the drawing itself (none of us are professional artists),

show and explain materials, doubts?

Ex.1: Body silhouette (20min)

1.1. No shoes and extra clothes or accessories that may disturb the silhouette drawing.

1.2. Think of a pose that represents you: who you are, what you do for a living, what you like to do, or

how you feel about having Leprosy (working, sleeping, dancing, cooking...).

1.3. Ask the participant to lie down in the desired posture and trace the silhouette of her body with a

pencil.

1.4. While the tracing is being done, ask questions to the participant to continue the rhythm of the

interview:

- How would you describe yourself before you had or knew you had Leprosy?

- Is it different now, in what way?

- What do you think has been the biggest change?

- How would you describe yourself right now?

- After the diagnosis of Leprosy disease, do you feel you know your disease?

1.5. After helping the participant to stand up, ask him/her to choose a colour for the silhouette that

best represents him/her (who he/she is, what he/she looks like).

1.6. Ask the participant to choose a colour for his/her hands: silhouette, print hands with gloves and

paint, etc.

Ex. 2: DIAGNOSIS OF LEPRA DISEASE (30 min)

2.1. Think about what symbols represent you how you were like before you knew you had Leprosy

and about how and where you would draw them on your body map.

2.2. Remember the moment you were diagnosed with Leprosy, how it affected you at that time, how

you are now and how you would reflect it in your body map.

2.3. Draw symbols or whatever you feel like, reflecting that period when you found out you had

Leprosy disease.

2.4. Encourage the participant to connect the drawings from before and after learning that he/she

had Leprosy (arrows, path, steps, etc.), to show the process.

HOMEWORK (5 min)

Hand in homework script to bring to the next session (symbol and personal message).

**SESSION 2: LIVING WITH LEPROSY DISEASE**.

Ex. 1: Personal symbol and slogan (10 min)

1.1. Ask the participant to show his/her symbol and slogan and explain them.

1.2. If the participant wants to cut and paste his/her symbol and slogan directly on her body map,

help him/her and do not forget to ask the meaning of the place where it will be put on the map.

1.3. If they are drafts, ask the participant to replicate a larger version on the body map or suggest

typing the message in a larger font size on a computer and printing it out.

1.4. If they have the symbol/image in mind, suggest looking it up on the internet and adding it to the

map in the next session. If the facilitator does it, don't forget to write down where you want to place

it.

1.5. Potential questions to guide the experience:

- What personal symbol and slogan have you chosen to describe your experience when you found out

you had Leprosy?

- Who are you as a person? What is your philosophy of life? What keeps you going?

- Can you explain the meaning of your symbol and slogan?

- Where do you want to place these symbols on your body map and why?

Ex. 2: Marks on/under the skin (35 min)

2.1. Ask the participant to brainstorm aspects that he/she would like to see represented around

his/her body (his/her life after learning that he/she had Leprosy, behaviour of his/her environment

since having Leprosy, relationships with others, relationship with the doctor/health personnel, etc.).

2.2. Then choose some (or all) aspects that emerged and ask the participant to draw or use symbols

to capture those experiences.

2.3. Ask the participant to visually review her body map from head to toe to identify specific marks.

These marks may be scars, past injuries, areas of stress or emotion. Ask the participant to think of the

"signs" or "marks" in a broad way: they can be on or under the skin (e.g., places where they have had

surgery, areas on or inside the body where they have experienced pain/illness/stress, nutrition and

diet, smoking, alcohol...).

2.4. Ask the participant to tell more about the marks, asking questions such as: Where did you get

that scar? How did it happen?

2.5. While drawing the symbols, ask the participant what he/she does to keep himself/herself

healthy/well. You should dwell on aspects mentioned in the interview.

Questions to guide the exercise:

- How has it affected you physically and emotionally?

- Has it affected your day-to-day life, in what way?

- Do you believe you are still fulfilling your duties?

- Has it affected your family relationships, your relationship in the community?

- If you look from head to toe at your body map, can you identify specific marks on your body that are

related to your health status before and now (before and after the diagnosis of Leprosy: Illness,

mental health, stress, treatments...)?

- How did those marks get on your body, what happened?

- In your daily life, what do you do to avoid getting sick? Do you use any kind of preventive measures

or natural remedies?

Notes: participants may not want to show all or any of their marks: RESPECT THIS and support the

process by encouraging them to describe the marks they do want. Drawing marks/scars may remind

them of traumatic experiences, so it is important to detect this in participants and offer them a pause

or, if needed, counselling.

Ex. 3: Self-portrait (10 min)

3.1. Ask the participant to think about who he/she is and what his/her face tells them.

3.2. Then, ask him to think about what image he/she gives to the world; how the world sees him/her.

3.3. Then, ask the participant to use these ideas to draw his/her face or to use symbols or clip-art

to represent certain aspects.

Potential questions to guide the exercise

We all have the same parts of our faces in more or less the same places (eyes, nose, mouth), but we

are different and our faces mean different things to different people.

- How would you like to represent your face, as it is, or in a more symbolic way?

HOMEWORK (5 min)

Prepare a message for the general public related to your current situation (e.g., person with Leprosy

disease, Leprosy patient, your experience with Leprosy).

**SESSION 3: ADAPTATION AND OVERCOMING**

Ex. 1: Message to others (5 min)

Capture a message that the participant would like to give to the general public about his

or her experience.

1.1. Ask the participant to read his or her message aloud and explain its meaning.

1.2. If the participant has not completed the task at home, give him/her a few minutes alone to think

about the message.

1.3. If necessary, help the participant to make the message concise.

1.4. If the participant wants to cut and paste his message directly on his map, help him/her and don't

forget to ask him/her about the meaning of where he/she puts it.

1.5. If possible and with the participant's approval, put a message larger than your version to make it

easier for others to read.

Potential questions to guide the exercise:

- After all that we have explored in these three sessions about your health and about Leprosy, I would

like you to think about your experience with Leprosy disease.

- What message would you want to give about your experience to people in general or to other

people with Leprosy disease? Why is this important for people to know?

- Where would you want to place this message on your body map?

Ex. 2: Body scanning (15 min)

Objective: To represent the impact of having Leprosy on social relations (on gender, stigma, access to

services, etc.); to locate and visualize the place/source of the participant's power and strength.

2.1. Ask and assist the participant to identify key experiences they have faced as a person with

Leprosy in relation to gender issues, discrimination, barriers to accessing health care or treatment,

etc.

2.2. Select key experiences to represent on their map and ask the participant to think of symbols or

images that capture those experiences.

2.3. Encourage participants to make connections between the drawings/images about their

experience with Leprosy (session 2) and their body (lines, arrows, etc.), and add symbols within this

trajectory.

2.4. Ask participants to think about where (in reference to their body and environment) they draw

the strength to overcome the challenges they have faced.

2.5. Help participants make connections between this area of personal strength or power and their

personal symbol or slogan (if relevant).

Potential questions to guide the exercise:

- We will now explore aspects of your social life, including your life as a man/woman, as a person

living in the countryside/city, your social relationships (partner, neighbours, friends, community),

and your use of health services.

- Have you ever faced difficulties in your social, spiritual, emotional life? What kind of difficulties

(Examples: fear, loss of hope, problems with partner, loss of faith, stigma, difficulty in accessing

diagnostic tests or treatment, etc.)?

- Now I want you to think about your strength and courage when you faced these problems. Where

do you get the strength? Where do you get the courage to keep going?

- Take a good look at your body map and concentrate on finding where that personal strength comes

from. Does it come from your arms? From your mind? Is it related to your personal slogan?

NOTE: It may be difficult for participants to think about the difficulties they have faced. One way to

get there is to try to make connections with what was said during the interview (pre-session 1).

Ex. 3: Supporting Structures (20 min)

Objective: to identify key people, institutions, organizations, or others (support structures, religion,

faith, etc.) that help the participant in his/her daily struggle.

3.1. Ask the participant to identify people or things that support him/her, and to choose a colour or

symbol that represents those supports.

3.2. Then ask the participant to explain how those people or things show their support. What do they

do to support you? What does it mean to feel supported?

3.3. If the participant chooses specific individuals, avoid using their real names on the body map: ask

the participant to choose a symbol or nickname to represent them.

Potential questions to guide the exercise:

- In this exercise I would like you to identify important (key) people, groups or things in your life that

support you or help you overcome some of the difficulties you face because you have Leprosy

disease.

- Who supports you? It can be an organization, a person (family member, professional, etc.), your

spirituality...

- How do these people show their support for you? What does this support mean to you?

NOTES: Participants may name specific people or organizations and want to capture this information

on their body map. It is important to remind them of the risk of being identified, and recommend

alternatives to using real identifiers (nicknames, colours or symbols). Some participants may not

know how to represent "support," so we may suggest using handprints as a general symbol of

support. If they decide to use this, it is best to use different colours for different types of support, or

to place these handprints in different areas of their map to show the different types of support they

receive.

Ex. 4: Drawing the future (10min)

Objective: to explore where participants are heading, their goals, and what they are striving for in

relation to their disease (Leprosy).

4.1. Ask the participant to think of a symbol or image that reflects what they are striving/struggling

for or what the future holds for them.

4.2. Ask him to draw this symbol in a place on his body map that represents the culmination

(attainment) of a goal, or what he is striving/striving for.

4.3. Ask the participant to choose a colour that represents achieving this goal/objective.

Potential questions to guide the exercise:

- Finally, I would like you to think about your future.

- What do you think will happen? Where do you think he/she will be/what will he/she be doing?

How do you think you will feel?

- How do you envision your future? What is your goal or dream?

- Where are you directing your efforts? It can be material, physical, emotional or spiritual.

PERSONAL NOTE: In this exercise it is advisable to choose the questions according to the participant's

personal history. While it is true that most of our participants are strong and hopeful to overcome

Leprosy, despair and the idea of death may appear here. We should be prepared in case a participant

collapses.

Ex. 5: Narrative (10 min)

Objective: to capture the participant's experience as he/she would like it to be told to others.

5.1. Encourage the participant to step back a little and look at his or her body map.

5.2. Ask the participant to tell his or her story using the map as a guide. Tell him/her that the purpose

of this exercise is for him/her to express how he/she would like others to see his/her life story.

Potential questions to guide the exercise:

- Now I would like you to look at your body map for a few minutes and think about what it tells you.

- Tell me, in a few words, about your experience with Leprosy disease through your body map and

how you would like this story to be remembered by others.

NOTE: Some participants may have a hard time with this because they have to remember the

meaning of the symbols they created during the first two sessions. To avoid losing information, help

participants by pointing out the key elements they should mention as they tell their story.

Ex. Final: Decorate/Finish (only if time permits)

F.1. Ask the participant to add any important details they wish to add to their body map and explain

why they have added those extra details.

F.2. Then ask the participant to draw links (e.g., lines, arrows) to refine the narrative/story that the

map should show.

F.3. Be sure to clarify any confusion in your narrative.

Potential questions to guide the exercise:

- The body map is almost complete. This is your last chance to add symbols or connections that you

think would be important to include in your story as a person with Leprosy disease.

- Is there anything missing? Is there anything you want me to add or change for you?

NOTE: It is very likely that there will not be enough time left for this. If possible, ask participants if

they want us to add or change symbols, colours, etc. for them. Write the instructions on a piece of

paper to do this another time.
