# Supplementary material for: Body map stories from Colombia: experiences of people affected by leprosy and the influence of peers during diagnosis and treatment
Source: Int J Equity Health. 2024 May 13;23:98. doi: 10.1186/s12939-024-02152-0 (PMC11092158; doi:10.1186/s12939-024-02152-0)
Supplement: Supplementary file 2 — Additional file 2: BMS Interview guide Spanish [file 12939_2024_2152_MOESM2_ESM.pdf]

ENTREVISTA PRE

Lectura y firma de consentimiento informado. Entrevista personal sobre su experiencia con la enfermedad de Lepra (35 min).

Preguntas para la entrevista semiestructurada:

- Me gustaría que me contara su experiencia con la enfermedad…
- ¿Usted prefiere que hablemos de la enfermedad como Lepra o como Hansen? ¿Por qué?
- ¿Sabía usted algo sobre esta enfermedad antes de saber que la tenía? ¿Qué pensaba de ella?
- ¿Quién le comunicó que tenía Lepra/ Hansen? ¿Cómo lo hizo? ¿Usted lo entendió? ¿Tuvo dudas? ¿Se las respondieron? ¿Siente que conoce su enfermedad?
- ¿Sabía en ese momento qué significaba tener la enfermedad?
- ¿Qué significó para usted el diagnóstico de la enfermedad? ¿Cómo le ha afectado físicamente? ¿Y emocionalmente?
- ¿Le ha afectado en el día a día? ¿En qué sentido?
- Dimensiones RBC: como le ha afectó su salud, su educación, su vida social/ familiar, su trabajo, su participación en la comunidad?
- ¿Cree que sigue cumpliendo con sus tareas?
- ¿Le ha afectado a sus relaciones familiares o su relación en la comunidad? ¿A quién le ha contado de su enfermedad?
- ¿Cómo se ha adaptado a su enfermedad?
- ¿Quién le ha apoyado para convivir con su enfermedad?
- ¿Qué recursos le han ayudado más?
- Ha tenido contacto con las asociaciones (Ej. Felehansen) u otros grupos de apoyo? ¡Cuéntenos de su experiencia!

SESIÓN 1: ANTES Y DESPUÉS DE SABER QUE TENGO LA ENFERMEDAD

<u>Introducción al BMS</u> (5 min)
importancia de lo que se cuenta, no del dibujo en sí (ninguno somos artistas profesionales), mostrar y explicar materiales, ¿dudas?

<u>Ej.1: SILUETA CORPORAL</u> (20min)
1.1. Fuera zapatos y ropa extra o accesorios que puedan molestar al dibujo de la silueta.
1.2. Piensa en una postura que te represente a ti: quién eres, en qué trabajas, qué te gusta hacer, o cómo te sientes teniendo Lepra. (trabajando, durmiendo, bailando, cocinando…).
1.3. Pedir a la participante que se tumbe en la postura deseada y trazar la silueta de su cuerpo con un lápiz.
1.4. Mientras se realiza el trazo, hacer preguntas a la participante para continuar el ritmo de la entrevista:

- ¿Cómo se describiría a usted mismo/a antes de tener o saber que tenía Lepra?

- ¿Es diferente ahora? ¿De qué manera?
- ¿Cuál cree que ha sido el mayor cambio?
- ¿Cómo se describiría ahora mismo?
- Después del diagnóstico de enfermedad de Lepra, ¿siente que conoce su enfermedad?

1.5. Tras ayudar a la participante a levantarse, pedirle que elija un color para la silueta que mejor la represente (quién es, cómo es).
1.6. Pedir a la participante que escoja un color para sus manos: silueta, imprimir manos con guantes y pintura…

Ej. 2: DIAGNÓSTICO DE LA ENFERMEDAD DE LEPRA (30 min)
2.1. Piensa qué símbolos y cómo y dónde los dibujarías en tu mapa corporal sobre cómo eras antes de saber que tenías Lepra.
2.2. Recuerde el momento en que le diagnosticaron Lepra, cómo le afectó en ese momento, cómo es usted ahora y cómo lo reflejaría en su mapa corporal.
2.3. Dibuje símbolos o lo que le apetezca, que refleje ese período en el que se enteró de que tenía la enfermedad de Lepra.
2.4. Animar al participante a conectar los dibujos de antes y después de saber que tenía Lepra (flechas, camino, pasos, etc.), para mostrar el proceso.

DEBERES PARA CASA (5 min)
Entregar guion de los deberes para traer la próxima sesión (símbolo y mensaje personal).

SESIÓN 2: VIVIR CON LA ENFERMEDAD DE LEPRA.

Ej. 1: Símbolo personal y eslogan (10 min)
1.1. Pedir a la participante que muestre su símbolo y eslogan y los explique.
1.2. Si la participante quiere cortar y pegar su símbolo y eslogan directamente en su mapa corporal, ayudarla y no olvidar preguntar el significado del lugar donde se va a colocar en su mapa.
1.3. Si son borradores, pedir a la participante que replique una versión de tamaño mayor sobre su mapa corporal o sugerir escribir el mensaje en un tamaño de letra mayor en un ordenador e imprimirlo.
1.4. Si tienen el símbolo/imagen en la mente, sugerir buscarlo en internet y añadirlo al mapa en la siguiente sesión. Si lo hace el facilitador, no olvidar apuntar dónde lo quiere colocar.
1.4. Preguntas potenciales para guiar la experiencia:
- ¿Qué símbolo personal y eslogan has escogido para describir tu experiencia al enterarte que tenías Lepra?
- ¿Quién eres tú como persona? ¿Cuál es tu filosofía de vida? ¿Qué te hace seguir adelante?
- ¿Puedes explicar el significado de tu símbolo y tu eslogan?
- ¿Dónde quieres colocar estos símbolos sobre tu mapa corporal y por qué?

Ej. 2: Marcas sobre/bajo la piel (35 min)
2.1. Pedir al participante una lluvia de ideas sobre los aspectos que le gustaría ver representados alrededor de su cuerpo (su vida tras saber que tenía Lepra, comportamiento de su entorno desde que tiene Lepra, relaciones con los demás, relación con el médico/personal sanitario, etc.)
2.2. Después, escoge algunos (o todos) aspectos que surgieron y pide a la participante que dibuje o use símbolos para capturar esas experiencias.
2.3. Pedir a la participante que repase visualmente su mapa corporal de la cabeza a los pies, para identificar marcas específicas. Estas marcas pueden ser cicatrices, heridas pasadas, áreas de estrés o emoción. Pide a la participante que piense en las "señales" o "marcas" de una forma amplia: pueden ser sobre o bajo la piel (p.ej. lugares donde han tenido alguna intervención quirúrgica, áreas sobre o dentro del cuerpo donde han experimentado dolor/enfermedad/estrés, nutrición y alimentación, tabaco, alcohol…).
2.4. Pedir a la participante que cuente más sobre las marcas, haciendo preguntas como: ¿Dónde te hiciste esa cicatriz? ¿Cómo pasó?
2.5. Mientras dibujáis los símbolos, preguntar a la participante qué hace para mantenerse sana/en buen estado. Habría que detenerse en aspectos mencionados en la entrevista.

Preguntas para guiar el ejercicio:

- ¿Cómo le ha afectado físicamente? ¿Y emocionalmente?
- ¿Le ha afectado en el día a día? ¿En qué sentido?
- ¿Cree que sigue cumpliendo con sus tareas?
- ¿Ha afectado a sus relaciones familiares? ¿A su relación en la comunidad?
- Si mira de la cabeza a los pies su mapa corporal, ¿puede identificar marcas específicas en su cuerpo que estén relacionadas con su estado de salud de antes y el de ahora? (antes y después del diagnóstico de Lepra: enfermedad, salud mental, estrés, tratamientos…).
- ¿Cómo llegaron esas marcas a su cuerpo, qué pasó?
- En su día a día, ¿qué hace para evitar ponerse enferma/o? ¿Usa algún tipo de medida preventiva, o remedios naturales?

Notas: los participantes pueden no querer mostrar todas o ninguna de sus marcas: RESPETAR ESTO y apoyar el proceso animándolos a describir las marcas que sí quieren. Dibujar las marcas/cicatrices puede recordarles experiencias traumáticas, por lo que es importante detectarlo en los participantes y ofrecerles una pausa o, si lo necesitan, counselling.

Ej. 3: Autorretrato (10 min)
3.1. Pedir al participante que piense sobre quién es él/ella y qué les dice su cara.
3.2. Después, pedirle que piense sobre qué imagen da al mundo; cómo le ve el mundo.
3.3. Después, pedir al participante que use esas ideas para dibujaren su cara o use símbolos o clip-art para representar ciertos aspectos.

Potenciales preguntas para guiar el ejercicio:

- Todos tenemos las mismas partes de la cara en los mismos sitios más o menos (ojos, nariz, boca), pero somos diferentes y nuestras caras significan diferentes cosas para diferentes personas.
- ¿Cómo le gustaría representar su cara? ¿Tal cual es? ¿O en una forma más simbólica?

DEBERES PARA CASA (5 min)
Preparar un mensaje para el público general relacionado con tu situación actual (p. ej: persona con enfermedad de Lepra, paciente con Lepra, tu experiencia con el Lepra).

SESIÓN 3: ADAPTACIÓN Y SUPERACIÓN

Ej. 1: Mensaje a los demás (5 min)
Capturar un mensaje que al participante le gustaría dar al público general sobre su experiencia.
1.1. Pedir al participante que lea su mensaje en voz alta y explique su significado.
1.2. Si el participante no ha completado la tarea en casa, darle unos minutos solo/a para que piense en el mensaje.
1.3. Si es necesario, ayudar al participante a hacer el mensaje conciso.
1.4. Si el participante quiere cortar y pegar su mensaje directamente en su mapa, ayudarle y no olvidar preguntarle sobre el significado de dónde lo coloca.
1.5. Si es posible y con la aprobación del participante, poner un mensaje más grande que su versión para que sea más fácil para los demás leerlo.

Preguntas potenciales para guiar el ejercicio:
- Después de todo lo que hemos explorado en estas tres sesiones sobre su salud y sobre el Lepra, me gustaría que pensara en su experiencia con la enfermedad de Lepra.
- ¿Qué mensaje querría dar sobre su experiencia a la gente en general o a otras personas con la enfermedad de Lepra? ¿Por qué es importante para la gente saber esto?
- ¿Dónde querría colocar este mensaje en su mapa corporal?

Ej. 2: Escaneado del cuerpo (15 min)
Objetivo: Representar el impacto de tener Lepra en las relaciones sociales (sobre género, estigma, acceso a servicios, etc.); localizar y visualizar el lugar/la fuente del poder y la fuerza del participante.
2.1. Pedir y ayudar al participante a identificar experiencias clave a las que haya tenido que enfrentarse como persona con Lepra en relación a asuntos de género, discriminación, barreras de acceso a atención sanitaria o tratamientos, etc.
2.2. Seleccionar experiencias clave para representar en su mapa y pedir al participante que piense en símbolos o imágenes que capturen dichas experiencias.
2.3. Animar a los participantes a hacer conexiones entre los dibujos/imágenes sobre su vivencia con el Lepra (sesión 2) y su cuerpo (líneas, flechas, etc.), y añadir símbolos dentro de esta trayectoria.
2.4. Pedir a los participantes que piensen de dónde (en referencia a su cuerpo y a su entorno) sacan la fuerza para superar los retos a los que se han enfrentado.

2.5. Ayudar a los participantes a hacer conexiones entre esta área de fuerza o poder personal y su símbolo o eslogan personal (si es relevante).

Preguntas potenciales para guiar el ejercicio:

- Ahora vamos a explorar aspectos de su vida social, incluyendo su vida como hombre/mujer, como persona que vive en el campo/ciudad, sus relaciones sociales (pareja, vecinos, amigos, comunidad), y su uso de servicios sanitarios.
- ¿Se ha enfrentado alguna vez a dificultades en su vida social, espiritual, emocional? ¿Qué tipo de dificultades? (ejemplos: miedo, pérdida de esperanza, problemas con la pareja, pérdida de fe, estigma, dificultad de acceso a pruebas diagnósticas o tratamientos, etc.)
- Ahora quiero que piense sobre su fuerza y coraje cuando se enfrentó a estos problemas. ¿De dónde saca la fuerza? ¿Dónde consigue el coraje para seguir adelante?
- Mire bien su mapa corporal y concéntrese en encontrar de dónde viene esa fuerza personal. ¿Viene de sus brazos? ¿De su mente? ¿Está relacionado con su eslogan personal?

NOTA: Puede ser difícil para los participantes pensar en las dificultades a las que se han enfrentado. Un medio para llegar ahí es tratar de hacer conexiones con lo que se dijo durante la entrevista (pre-sesión 1).

Ej. 3: Estructuras de apoyo (20 min)
Objetivo: identificar personas clave, instituciones, organizaciones u otros (estructuras de apoyo, religión, fe, etc.) que ayuden al participante en su lucha de cada día.

3.1. Pedir al participante que identifique personas o cosas que le apoyan, y que elija un color o símbolo que represente esos apoyos.
3.2. Después, pedir al participante que explique cómo esas personas o cosas muestran su apoyo. ¿Qué hacen para apoyarle? ¿Qué significa sentirse apoyado?
3.3. Si el participante escoge a individuos específicos, evitar usar sus nombres reales en el mapa corporal: pedir al participante que escoja un símbolo o seudónimo (*nickname*) para representarles.

Preguntas potenciales para guiar el ejercicio:

- En este ejercicio me gustaría que identificase a personas importantes (clave), grupos o cosas en su vida que le apoyan o le ayudan a superar algunas de las dificultades a las que se enfrenta por tener la enfermedad de Lepra.
- ¿Quién le da apoyo? Puede ser una organización, una persona (familiar, profesional, etc.), su espiritualidad…
- ¿Cómo muestran estas personas su apoyo hacia usted? ¿Qué significa este apoyo para usted?

NOTAS: Los participantes pueden nombrar personas específicas u organizaciones y querer plasmar esta información en su mapa corporal. Es importante recordarles el

riesgo de ser identificados, y recomendarles alternativas a usar identificadores reales (*nicknames*, colores o símbolos).
Algunos participantes pueden no saber cómo representar “apoyo”, así que podemos sugerir usar huellas de manos como un símbolo general de apoyo. Si deciden usar esto, mejor usar diferentes colores para diferentes tipos de apoyo, o situar estas huellas en diferentes áreas de su mapa para mostrar los diferentes tipos de apoyo que reciben.
Ej. 4: <u>Dibujando el futuro</u> (10min)
Objetivo: explorar hacia dónde se dirigen los participantes, sus metas, y por qué se esfuerzan en relación con su enfermedad (Lepra).

4.1. Pedir al participante que piense en un símbolo o imagen que refleje por lo que se están esforzando/luchando o que les depara el futuro.
4.2. Pedirle que dibuje este símbolo en un lugar de su mapa corporal que represente la culminación (el alcance) de una meta, o por lo que está luchando/esforzándose.
4.3. Pedir al participante que elija un color que represente conseguir esta meta/objetivo.

<u>Preguntas potenciales para guiar el ejercicio</u>:

- Finalmente, me gustaría que pensase en su futuro.
- ¿Qué cree que pasará? ¿Dónde cree que estará/qué estará haciendo? ¿Cómo cree que se sentirá?
- ¿Cómo imagina su futuro? ¿Cuál es su objetivo o su sueño?
- ¿Hacia dónde dirige sus esfuerzos? Puede ser algo material, físico, emocional o espiritual.

NOTA PERSONAL: en este ejercicio conviene escoger bien las preguntas según la historia personal del participante. Si bien es cierto que la mayoría de nuestros participantes se muestran fuertes y esperanzados para superar el Lepra, aquí pueden aparecer la desesperanza y la idea de la muerte. Deberemos estar preparados por si algún participante se derrumba.

<u>Ej. 5: Narrativa</u> (10 min)

Objetivo: **capturar la experiencia del participante tal y como a él/ella le gustaría que fuese contada a otros**.

5.1. Animar al participante a alejarse un poco y mirar su mapa corporal.
5.2. Pedir al participante que nos cuente su historia usando el mapa como una guía. Decirle que el propósito de este ejercicio es que exprese cómo le gustaría que otros vieran la historia de su vida.

<u>Preguntas potenciales para guiar el ejercicio:</u>

- Ahora me gustaría que mirase unos minutos su mapa corporal y piense qué le dice.
- Cuénteme, en unas cuantas palabras, su experiencia con la enfermedad de Lepra a través de su mapa corporal y cómo le gustaría que esta historia fuese recordada por otras personas.

NOTA: A algunos participantes les puede costar mucho esto porque tienen que recordar el significado de los símbolos que crearon durante las primeras dos sesiones.

Para no perder información, ayudar a los participantes señalando los elementos clave que deberían mencionar mientras narran su historia.

Ej. Final: Decorar/Terminar (sólo si el tiempo lo permite)

F.1. Pedir al participante que añada cualquier detalle importante que desee a su mapa corporal y que expliqué por qué ha añadido esos detalles extra.
F.2. Después, pedir al participante que dibuje enlaces (p. ej. líneas, flechas) para refinar la narrativa/historia que el mapa debería mostrar.
F.3. Asegurarse de aclarar cualquier confusión en su narrativa.

Preguntas potenciales para guiar el ejercicio:

- El mapa corporal está prácticamente terminado. Esta es su última oportunidad para añadir símbolos o conexiones que usted cree que sería importante incluir en su historia como persona con la enfermedad de Lepra.
- ¿Falta algo? ¿Hay algo que quiere que yo añada o cambie por usted?

NOTA: Es muy probable que no quede suficiente tiempo para esto. Si es posible, preguntar a los participantes si quieren que añadamos o cambiemos símbolos, colores, etc. por ellos. Escribir las instrucciones en un papel para hacerlo en otra ocasión.
