# Supplementary material for: Body map stories from Colombia: experiences of people affected by leprosy and the influence of peers during diagnosis and treatment
Source: Int J Equity Health. 2024 May 13;23:98. doi: 10.1186/s12939-024-02152-0 (PMC11092158; doi:10.1186/s12939-024-02152-0)
Supplement: Supplementary file 3 — Additional file 3: Additional Verbatims English translation [file 12939_2024_2152_MOESM3_ESM.docx]

Additional file 3: Additional Verbatims English translation

| VS1: | *One was so naive. I didn't go to the doctor then, for me it was like the flu that gave me fever and shiver, so I drank aguapanela with lemon and that was it. (Leticia)* |
| --- | --- |
| VS2: | *But I went to the pharmacy, I told the man who attended me to give me a good cream for fungus. It was a bit expensive, and I started to apply it, but it didn't work, I got more plaques. (Yamileth)* |
| VS3: | *It took more than 2 years, because sometimes I was reluctant to take the exam, because I said the journey is far away or going to (name of a municipality with health care specialised in leprosy) is farther away. (Mariposita)* |
| VS4: | *When I was at the aunt's house, the cousin said directly into my ear, "It seems to be leprosy". My aunt, who conveniently had a very close relationship with the doctors because she was known for her family and for being a very, very wealthy person, referred me to a doctor who was the eminence of dermatology in the department at that time. So, she picked up the phone and called him (...), "I have my nephew in this situation. Can you take care of him? He's very busy." When she hung up the phone, she picked up $100. 000. We are talking about 2006, she picked up $100,000 and told me that “he is not charging me, he is waiting for you”. (Luchador incansable)* |
| VS5: | *There are a lot of doctors who don't know what leprosy is, or if they know what it is, they have never been in touch with the treatment or anything. (Chamo)* |
| VS6: | *At my new EPS (empresa prestadora de servicios, Colombian health inssurance) they started to do the logistics to get the therapy, but nobody knew the disease, nothing, nobody knew anything about it. So, everybody started to learn with me. (Angie)* |
| VS7: | *They explained to me that the disease can be confused with other diseases such as sugar and nerve problems. (Flor del campo)* |
| VS8: | *At the same time, I think that if they (HCW) had been more careful, my hand would not have turned like this. (Flor del campo)* |
| VS9: | *I went to a general practice appointment and was alone with the doctor, God bless her, I went in and she said to me, "What do you have?". Then I told her I had something on my knee, to which she yelled at me and told me to pull my pants up. She said to me, "You have cancer.". She didn't even touch me or look closer, and suddenly she said to me, "You have a cancer there, you have to go…” I don´t know where, so I left in a bad mood thinking: “How can this woman be so rude to patients?” (Mariposita)* |
| VS10: | *And after that, about two years later, the doctors ordered further examinations because I became very thin. I felt very bad and contacted every doctor and they did not know anything. Two months after my son was born, I swelled up all over and when I started to look for a doctor, they gave me a lot of appointments. (Hela)* |
| VS11: | *I had already lost my strength, everything fell out of my hands, I could no longer hold anything with my hands and all of this distressed me. (Flor del campo)* |
| VS12: | *I remember my mum putting an epilator up my nose when my nose was blocked and getting everything out. So, I took the epilator and put it in my nose, it got everything out and I felt reassured because I said to myself: now I can breathe again. And the amount of blood, well, I just swallowed it. (Lazaro)* |
| VS13: | *The neurologist told me: “You are very bad off in life, because this is what old people get. But you are the first girl, but you are young, you are very bad off”, and I thought: “Well, thank you”. (Maria)* |
| VS14: | *As soon as I went in, the doctor told me: “Please, the next time you come, take a bath.” So, it wasn't the first time they told me to take a bath, they had told me that before. (Lazaro)* |
| VS15: | *Because the biologicals (for arthritis) are strong (…), because the biologicals lower one's defences (…) Those are such a strong treatment that you feel, because you don't know if you are dying, because you can have a heart attack, a cardiac arrest and respiratory arrest (...). You arrive there and it is like arriving at the slaughterhouse, you don't know if you leave alive or dead, and if you leave alive, you leave deader than alive, you don't even feel your legs. You have to carry food for the whole day, sometimes the whole day, sometimes two hours, it depends on the treatment that you get (...) injected intravenously, and that is very slow because it is so poisonous that it is tenacious. And that made me develop leprosy. (Mariposita)* |
| VS16: | *When she (his wife) found out, she went to spread the story that I had HIV, and she told me: “I can't live with you anymore. I want you to leave”. At that time, I was living with my in-laws, and they kicked me out of the house, they took me out like taking a dog out of a church and they threw me out onto the street. Well, that house was not mine, I had absolutely no support from anyone, so I said: “I am going to become a street dweller”. (Lazaro)* |
| VS17: | *My mother went to XX(name of town) and had the tests done and they came out positive and they sent her to the EPS. She started her regular treatment and when she was about to finish the treatment, the people from the German association came here to do a sensitivity test and that is when (...) I had no sensitivity in one part of my arm. (Maria)* |
| VS18: | *Thank God the dermatologist had worked with people with Hansen´s disease. (Angie)* |
| VS19: | *First, they did a lot of studies to see if it was a skin cancer or if it was a blood cancer when they did the first biopsy. From there, after about two, three months of studying, studying and studying the biopsy, the bacteriologist suggested that they do a skin smear to detect if it was leprosy. (Saray)* |
| VS20: | *I went to a dermatologist, but he said I had nothing, so two years passed again until my legs started to look like raisins. Then I said: "no", that's when I got scared and I went to a dermatologist, I said: "I can't wait any longer because it's getting worse." (Angie)* |
| VS21: | *I didn't feel anything in my arm, I pinched myself and didn't feel anything and the arm started to feel hot, like I had it in an oven. Then my husband was very insistent that I go to the doctor, but I pretended not to notice and told him I didn't have anything.... When my mother-in-law was home, she asked me why I hadn't gone to the doctor. My mother-in-law worked at the sanatorium and told a doctor about it. (Flor del campo)* |
| VS22: | *A friend told me: “Go to a church because my God is going to heal you.”, but I was sure that the treatment was going to heal me. (Lazaro)* |
| VS23: | *Well, I took it (the medication) every day at the same time, not a minute more, not a minute less, it was always on time. (Palomita)* |
| VS24: | *Well, I say that it´s not only receiving treatment, I think that everything is important: being in that place, the sanatorium.... I think it is about all the things because you can't say it´s just the one thing, so I think that everything helps you well. (el Negro)* |
| VS25: | *People are very reckless and honest and because of the effect of clofazimine, people come to ask you what you have, and I wouldn't want anyone to ask because I am ashamed (...) I say that for women it is more difficult because you are vainer, and they tell you that you are strange and ugly, so this is terrible. (Angie)* |
| VS26: | *One time, one man told me: “Excuse me mommy, now they sell some powders, which help to cover stains.” And I started crying, because a man telling one this, this is very hard (...) he was a stranger, a very effusive man. (Angie)* |
| VS27: | *One gets scared when they give you the first treatments, the first pills. They even change your urine, right, so you say: “My God, what are these people giving me, I'm urinating a different colour!”, right? (Chamo)* |
| VS28: | *My liver did not accept the treatment well (...), I lost weight, there are also times when I have no energy (...). They took the tests, and my bilirubin levels were almost five times higher than normal, and the doctor told me that the levels were very high, and I was worried (...). Sometimes I don't look so red but rather yellow. (María)* |
| VS29: | *It took about three years until one day I was told that I was cured. Until this time, I didn't know if I was going to be cured or not. (Daniel)* |
| VS30: | *Well, I was there with the doctor, up and down with the doctor, fighting and taking my medicine. (Mariposita)* |
| VS31: | *So that was very frustrating for me because I was thinking that the treatment was going to last two years and that I was going to stay well, but when I got to the two years, they told me that I had to start all over again. I felt like I was never going to finish with it (...). I went into depression, I didn't want to take any more pills, I said that I wanted to stay like this, that it was God's will, that if he wanted to heal me, he would heal me, that if he wanted to take me, he would take me. (Saray)* |
| VS32: | *But now and then, I stopped taking the treatment they were giving me, they never explained to me what it was about, I knew what it was, but they didn't tell me what it was about (...) I didn't know what it was about or what was going to happen. So, since I felt better and I didn't feel anything, I was working as a painter, my colleagues would tell me to have a beer and I forgot about the pills. But when I felt bad again, I would go back and they (HCW) would pull my ears, but they never explained to me what was going to happen to me (....) if they had informed me, I would have taken my pills, at that time they didn't tell you that it was going to cure you or anything. At that time, I didn't know what was going to cure the disease, only that the pain was going to go away. (Carlos)* |
| VS33: | *At least, I say that the health department makes a mistake when they tell you to go to the nutritionist. They tell you that you must eat chicken breast, eat well, I don't know what, but it's a lie because if you don't have the resources to eat what they say, you eat what you can. But the food, I think, influences too much. (Angie)* |
| VS34: | *A lady said to me: "What do you have?”. And I told her; “I don't know, I went to the doctors, and they haven't given me a reason for this illness”. She told me: “Why don't you go to this man? He can tell you what he has. Maybe you have the same problem that he has”, so I went to the man, and he immediately told me calmly that he is going to give me the number of a doctor. (el Negro)* |
| VS35: | *Yes, because I don't want any of them to get the disease or to be diagnosed too late, so I have tried to share information about the disease. (Yamileth)* |
| VS36: | *I was very responsible, I took the pills, like my daughter who is very judicious with the treatment, well, to cure us because we know that it is a very tenacious illness, I know the case of my cousins, they had to wash their hands, their feet, they had no feet. (Mariposita)* |
| VS37: | *In my family, the husband of one of my mother's sisters, he got the disease many years ago and he took the treatment and was cured and everything was fine. When my mother called my aunt to tell her what had happened, the diagnosis was positive, he came here and brought me some brochures he had, that talked about the disease, the care of the hands, the care of the feet, of the eyesight, then he came and started to explain and he named me the doctor (...), he told me: “a doctor called (...) was going to see you, that doctor is very good.”, he started to explain to me: “Don't worry, it's nothing bad, you're going to be cured, you're going to do such and such, you take your treatment, you take all the right things, don't drink liquor, don't smoke!” He explained everything to me. And that was like my encouragement because he gave me that light. (Saray)* |
