# Supplementary material for: Body map stories from Colombia: experiences of people affected by leprosy and the influence of peers during diagnosis and treatment
Source: Int J Equity Health. 2024 May 13;23:98. doi: 10.1186/s12939-024-02152-0 (PMC11092158; doi:10.1186/s12939-024-02152-0)
Supplement: Supplementary file 4 — Additional file 4: Additional Verbatims Spanish [file 12939_2024_2152_MOESM4_ESM.docx]

Additional file 4: Additional Verbatims Spanish

| VS1: | *Así uno inocente y tal vez, no sé Dios mío, en ese entonces yo no iba ni al médico, para mí era como una gripe que me dio fiebre, escalofrío. Me tomo una aguapanela con limón y ya (Leticia)* |
| --- | --- |
| VS2: | *Me fueron saliendo más honguitos, pero yo fui a la farmacia. Le dije al señor que me atendió, que me diera una cremita buena para hongos. Salió un poco costoso y empecé a aplicarla, pero no me servía, me salían más plaquitas. (Yamileth)* |
| VS3: | *Pasó más de 2 años, porque yo me resistía a veces que me hicieran el examen. Porque yo decía: el viaje es lejos o ir a XX (nobre de cuidad con servicios especialicados en lepra) es más lejos. (Mariposita)* |
| VS4: | *Cuando estuve en la casa de la tía y el primo me dijo, directamente al oído me dijo “parece ser lepra”. Entonces ella me direccionó, prácticamente ella tenía una relación muy estrecha con los médicos porque en esa familia... y pues también para una persona muy muy pudiente conocida, por un médico en ese momento era la eminencia dermatológica en el departamento. Al teléfono lo llamó, (…) “tengo a mi sobrino en esta situación, ¿usted me lo puede atender? está muy ocupado” Cómo son las conversaciones con el aire. Cuando colgó el teléfono, sacó $100.000 estamos hablando del año en agosto del 2006, sacó $100.000 y me dijo que “él a mí no me cobra. Vaya que lo está esperando”. (Luchador incansable)* |
| VS5: | *Hay mucho médico que no sabe qué es lepra, o si sabe qué es, nunca le he tocado el tratamiento ni nada. (Chamo)* |
| VS6: | *Ya en la nueva EPS (empresa prestadora de servicios) empezar a tramitar lo del medicamento, pero iguales a ninguno sabía de la enfermedad y nada, nadie sabía. Entonces todos empezaron a aprender conmigo. (Angie)* |
| VS7: | *Ellos me explicaron la enfermedad se puede mezclar con otras enfermedades que puede ser azúcar, problemas de los nervios. (Flor del campo)* |
| VS8: | *Al mismo tiempo pienso que, si a mí me hubieran puesto más cuidado no me hubiera ... no se me hubiera vuelto la mano así. (Flor del campo)* |
| VS9: | *Fui a cita medicina general, y me tocaba en la soledad con la doctora que Dios la guarde. Entré y me dijo ¿qué es lo que usted tiene? Entonces yo le dije: “Es que me salió algo en la rodilla”, y me decía gritando “levantes el pantalón”, y me dijo “usted tiene cáncer”, así de una vez me dijo ni me tocó, ni se me acerco ni nada, y así me dijo “usted tiene un cáncer ahí, y tiene que ir a no sé qué” entonces yo salí con mal genio de ahí, y yo pensaba en qué momento esta señora es tan grosera con el paciente. (Mariposita)* |
| VS10: | *Y después de eso como 2 años más. Entonces los médicos me mandaron otros exámenes, porque yo me puse muy delgada. Yo me sentía muy mal y yo caminaba en cada médico y ellos no sabían nada, a los 2 meses de estar mi hijo de nacido yo me hinché toda, yo cuando empecé a buscar al médico me mandaron a muchas citas. (Hela)* |
| VS11: | *Que ya había perdido la fuerza que ya todo se me caía yo ya no sostenida nada en la mano, y todo eso me angustió. (Flor del campo)* |
| VS12: | *Comenzó a taparse la nariz yo recuerdo que mi mamá cuando se me tapa la nariz se metió un depilador y sacaba las carachitas. Entonces yo me metí el depilador y todo lo que cogía lo jalaba. Entonces por eso me acaba con la nariz, pero yo sentía tranquilidad porque decía yo ya puedo respirar, y ese montón de sangre pues me la tragaba. (Lazaro)* |
| VS13: | *La neuróloga me decía “tú eres muy de malas en la vida, porque esto le dan a los viejitos, pero tú eres la primera chica, pero tú tienes 24 años, eres muy de malas”, y yo” bueno, gracias” (Maria)* |
| VS14: | *Apenas entré la doctora me dijo “por favor la próxima vez que venga báñese”. Entonces no era la segunda vez que me decían báñese, ya antes me lo habían dicho (Lazaro)* |
| VS15: | *Porque los biológicos (para la artritis) están fuerte (...) porque el biológico le baja a uno las defensas (...) un tratamiento tan fuerte que usted se siente, porque usted no sabe si se muere se le iba porque puede darle un infarto cardíaco, un paro cardíaco y paro respiratorio (…) Usted llega, es cómo llegar al matadero, no sabes si sale viva o si sale muerto de ahí, y si sale viva sale más muerta que viva, usted no siente ni las piernas. Usted tiene que llevar comida para comer todo el día allá. A veces todo el día a veces me decía a veces dos horas, depende del tratamiento que les pongan (…) inyectados en intravenosos. Y eso es súper lento, porque es algo tan venenoso qué es tenaz. Y eso hizo que se me desarrollara a mí la Lepra. (Mariposita)* |
| VS16: | *Cuando ella (esposa) se enteró, fue a regar el cuento que yo tenía VIH. Y ella me dijo “yo no puedo vivir más con usted. Yo quiero que usted se vaya”. En ese tiempo yo vivía con mis suegros, y me sacaron de ella y me sacaron cómo sacar un perrito de una iglesia pues no a patadas pero si fuera, y me echaron para la calle. Pues esa casa no era mía, no tuvo el apoyo absolutamente de nadie, entonces yo dije me voy a convertir en un habitante de la calle. (Lazaro)* |
| VS17: | *Mi mamá fue a XX (nombre de cuidad), se hizo los exámenes y le salieron positivos, ya, ya, ya mandaron como toda secretaría y secretaria a la EPS. Ella empezó su tratamiento normal, tú y yo. Cuando iba a finalizar el tratamiento, pues fueron de aquí los de la asociación alemana para hacer como un test de sensibilidad y ahí fue cuando pues (…) me hizo un test y yo no tenía sensibilidad en una parte del brazo. (Maria)* |
| VS18: | *Gracias a Dios al dermatólogo había trabajado con personas con Hansen. (Angie)* |
| VS19: | *Primero me hicieron muchos estudios para ver si era un cáncer de piel, si era un cáncer de sangre, cuando me hicieron la primera biopsia. De allí, el bacteriólogo, después de como dos, tres meses de estar en estudio, y estudio, y estudio a la biopsia sugirió que me hicieran una baciloscopia para detectar si era lepra (Saray)* |
| VS20: | *Me iba a mirar el dermatólogo, yo no tenía nada, entonces dure 2 años en eso. Después de que mis piernas empezaron a poner como una uva pasa, entonces yo dije..”No” ahí sí me asusté y me fui a la (XX nombre de cuidad), a un dermatólogo, yo dije “no, ya no puedo esperar más, porque se está avanzando”. (Angie)* |
| VS21: | *Yo no sentía nada en el brazo, yo me pellizcaba y no sentía y empezó como si lo tuviera en un horno sentía calor. Entonces mi esposo me insistía mucho y yo me hacía la disimulada y yo le decía que yo no tenía eso, los síntomas empezaron así... Cuando fue que mi suegra ya en mi casa me dijo que, porque no iba al médico, mi suegra trabajó en el sanatorio y ella por medio de ella le comentó a un médico. (Flor del campo)* |
| VS22: | *Porque un amigo me dijo, “vaya a una iglesia porque mi Dios lo va a sanar”, pero yo tenía claro que el tratamiento me iba a sanar. (Lazaro)* |
| VS23: | *O sea yo me lo tomaba todos los días a la misma hora, no pasaba ni un minuto más ni uno menos siempre era la hora. (Palomita)* |
| VS24: | *Pues yo digo que reciben el tratamiento, yo creo que todo, todo el estar en uno en ese lugar, el sanatorio, recibir todas... Yo creo que todas las cosas, le ayudan a uno bien, porque uno no puede decir, obtener exclusivo una sola cosa entonces yo creo que todo le sirve uno para bien. (el Negro)* |
| VS25: | *La gente es muy imprudente, entonces honestas y con el efecto de la clofazimina (...). Entonces la gente llega a preguntarle “¿usted qué tiene?”, y no quisiera que nadie le preguntara a uno, porque la vergüenza (…) yo digo que la mujer, es más difícil porque uno es como más vanidosa, y que le digan a uno que usted se vé todo extraño, toda fea. Entonces eso es terrible. (Angie)* |
| VS26: | *Una vez, me dijo “Disculpa mami” no sé qué, un hombre me dice “ahora venden unos polvos, qué le ayudan a tapar las manchas” no sé qué, y yo me suelto a llorar, porque que un hombre le dijera uno de eso es muy duro (…) era un desconocido, un hombre muy efusivo. (Angie)* |
| VS27: | *Uno si se asusta es cuando le dan los primeros tratamientos las primeras pastas, entonces que le cambie hasta la orina a uno cierto, entonces uno dice “Dios mío qué es lo que esta gente me está dando, yo estoy orinando de otro color!”, ¿cierto? (Chamo)* |
| VS28: | *Mi hígado tampoco lo ha aceptado muy bien (…), bajé de peso, también hay épocas en que la energía no es tan alta (…), me tomaron los exámenes y las bilirrubinas me salieron casi 5 y la doctora me dijo que estaba muy alto y estaba preocupada, (…) y que a veces no me veo tan roja sino más bien amarilla. (María)* |
| VS29: | *Hasta que un día me dijeron que yo ya estaba curado pasaron como 3 años. En ese tiempo, yo no sabía si me iba a curar o no. (Daniel)* |
| VS30: | *Pues allá con el médico, para arriba y para abajo con el médico, lucha y póngame medicamentos y hágame causas. (Mariposita)* |
| VS31: | *Deciden comenzar mi tratamiento de nuevo. Entonces eso fue muy frustrante para mí, porque yo venía mentalizada de que el tratamiento iba a ser dos años, que yo me iba a quedar bien pero yo llegué a los 2 años y me dijeron tienes que volver a comenzar. Yo me sentí, como que nunca iba a acabar con eso (…) yo entré en depresión, yo no quería tomarme más pastas. Yo decía que me quería quedar así, que ya fuera la voluntad de Dios, que si él me quería sanar que me sanara, que se me quería llevar, que me llevara. (Saray)* |
| VS32: | *Pero entonces a cada ratico yo he dejado de tomarme los tratamientos que me estaban dando. A mí nunca me explicaron de qué se trataba, yo sabía que era eso, pero no me contaron de qué se trataba (…) no sabía de qué se trataba ni que iba a suceder. Entonces yo como más me sentía mejor ya no, yo como trabajan pinturas y obras, entonces los compañeros me decían “Échate una cerveza” y yo no volvía a tomar los remedios. Y cuando me sentía mal yo volvía y me pegaban unos jalones de orejas, pero como uno nunca le explicaban a uno que le iba a suceder (…). Si a mí me hubieran informado, yo me tomaría mis remedios. En ese momento uno no le decía que se va a curar ni nada, es como ahorita que uno le da unas pastas para que le pase el dolor y listo. En ese tiempo yo no sabía qué se iba a curar la enfermedad, solamente que se iba a pasar el dolor. (Carlos)* |
| VS33: | *Por lo menos yo digo que la secretaría de salud cometen un error cuando le dicen “vaya a nutricionista”, que no sé qué. Ellos le dice “no se tiene que comer pechuga, alimentarse bien” no sé qué. Pero es mentira, porque si no tiene los recursos para comer lo que unos dicen, uno come lo que uno puede. La alimentación yo creo que influye demasiado. (Angie)* |
| VS34: | *Una señora me dijo qué, me dijo” ¿Tú qué tienes? “ Y yo le dije “no sé, voy donde los médicos y no me han dado razón de esta enfermedad”. Ella me dijo “porque no vas y me menciona el nombre del muchacho del señor... Él te puede decir que tienes , porque de pronto tú puedes tener ese mismo problema que él tiene”. Entonces yo fui donde el señor. Entonces él inmediatamente que me dijo “tú tienes eso, quiero en ese momento que estes tranquilo. Yo le voy a dar el número del doctor XX (nombre del doctor)” (el Negro)* |
| VS35: | *Si, porque no quiero que a ninguno de ellos les de la enfermedad o que tengan de pronto un diagnóstico muy tarde, entonces he tratado de compartir la información acerca de la enfermedad. (Yamileth)* |
| VS36: | *Si, fui muy juiciosa, me tomé las pastillitas, como mi hija que es muy juiciosa con el tratamiento, pues para curarnos, porque uno sabe que es una enfermedad muy tenaz, que yo conozco el caso de mis primos que les tocaba lavar los pies, las manitas, ellos estaban sin pies y sin piecitos. (Mariposita)* |
| VS37: | *en mi familia el esposo de una de las hermanas de mi mamá, a él le dió hace muchos años. Le dió la enfermedad y él se tomó el tratamiento y el curó y todo bien. Cuando mi mamá llamó a mi tía a contarle lo que le habían diagnósticado, me había salido positivo, él de una se vino para acá y me trajo unos folleticos que él tenía, que hablaban de la enfermedad, el cuidado de las manos, el cuidado de los pies, de la vista, entonces él se vino y se agarró a explicar, entonces me nombró a la doctora (…), me dijo allá lo va a ver una doctora que se llama (…), esa doctora es muy buena. Y él se agarró a explicarme: no se preocupe, eso no es nada malo, usted se va a curar, usted tal cosa, usted se toma su tratamiento, usted se toma todo juiciocita, no tome licor, no vaya a fumar, él se agarró a explicarme todo. Y ese fue como el aliento mío, porque él ya me dio como esa luz. (Saray)* |
