# Supplementary figures and images for: Body map stories from Colombia: experiences of people affected by leprosy and the influence of peers during diagnosis and treatment
Source: Int J Equity Health. 2024 May 13;23:98. doi: 10.1186/s12939-024-02152-0 (PMC11092158; doi:10.1186/s12939-024-02152-0)

## Slide 1
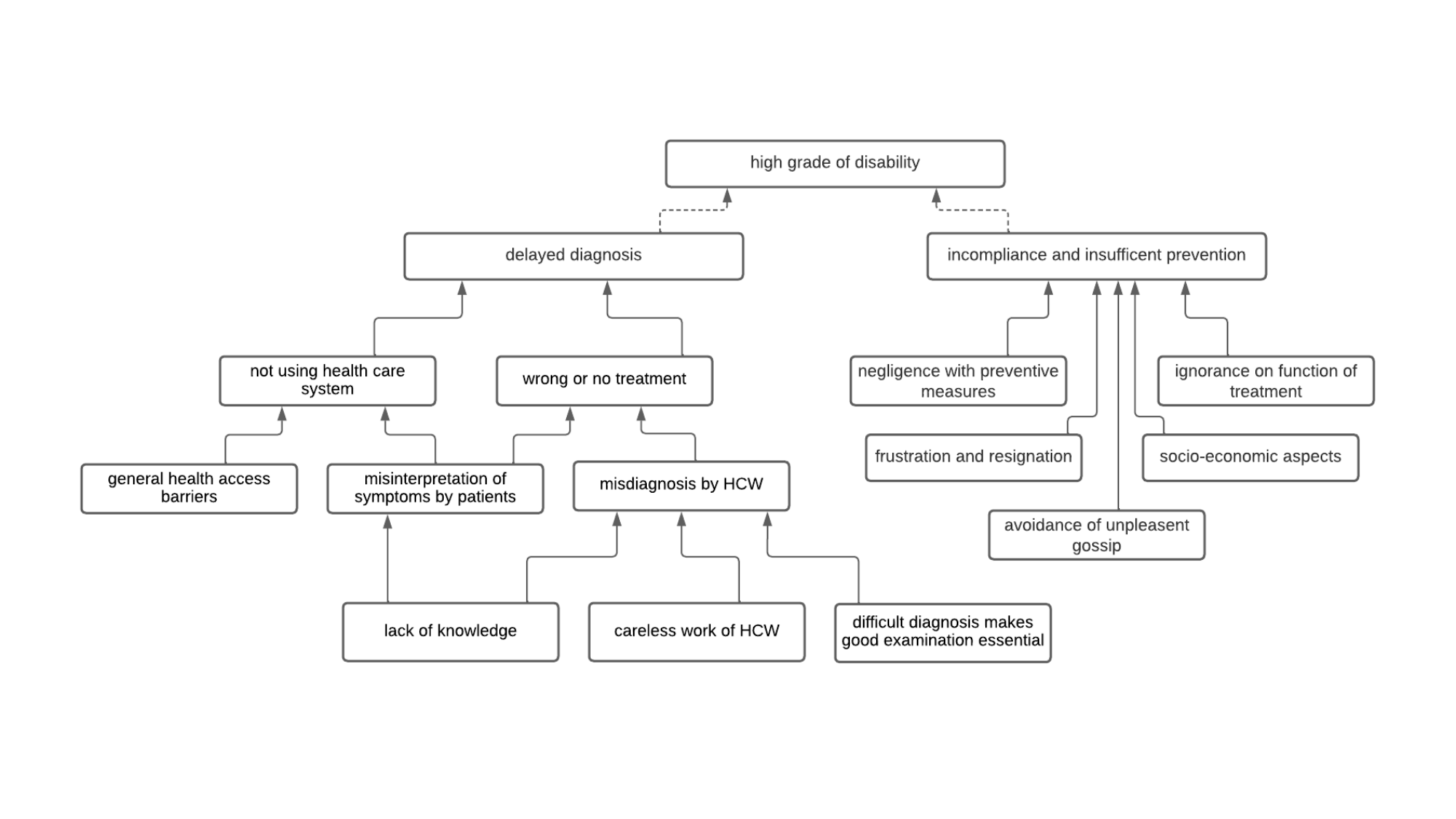

Supplement: Supplementary file 5 — Additional file 5: Initial thematic map exploring underlying phenomena of disability [file 12939_2024_2152_MOESM5_ESM.pptx]
